# Quality and quantity of data used by Health Canada in approving new drugs

**DOI:** 10.3389/fmed.2023.1299239

**Published:** 2023-11-30

**Authors:** Joel Lexchin

**Affiliations:** ^1^School of Health Policy and Management, York University, Toronto, ON, Canada; ^2^Department of Family and Community Medicine, University of Toronto, Toronto, ON, Canada

**Keywords:** Health Canada, drug regulation, orphan drugs, oncology drugs, clinical trials, patient demographics

## Abstract

**Background:**

This study examined multiple aspects about the approval of new drugs: the characteristics of the drugs, the quality and quantity of information that Health Canada discloses about the demographics of patients enrolled in clinical trials, the characteristics of the trial, and the type of review that it uses. It examines whether there have been changes in these measures between 1 September 2012 and 31 March 2022.

**Methods:**

A list of all new drugs approved, type of review used, and drug characteristics was generated from Health Canada annual reports. Therapeutic categories were identified from the World Health Organization Collaborating Center for Drugs Statistics Methodology. The Summary Basis of Decision documents of Health Canada were used to identify patient demographics in clinical trials and clinical trial characteristics.

**Results:**

Health Canada approved 326 new drugs for 407 indications. The percent of orphan drugs approved increased from 35.6 to 51.3%. The number of indications per drug decreased (*p* = 0.0817) as did the number of pivotal trials per drug (*p* = 0.0091). The percent of Phase 3 trials dropped from 76.3% in 2012–2015 to 64.8% in 2019–2022 (*p* = 0.005). There was also a statistically significant decrease in the percent of trials that were randomized, controlled, and blinded. The clinical trial characteristics of orphan drugs and the type of review used were both significantly different compared with non-orphan drugs. The percent of trials which had information about the number of patients enrolled, the percent of trials that provided the age of the patients, and the sex breakdown all significantly increased.

**Conclusion:**

The results show that there has been a change in regulatory standards that may be due to them becoming less rigorous, because of an adaptation to the number of orphan drugs being submitted or a combination of both reasons. At the same time, there has been some improvement in the transparency of data. Health Canada has recently embarked on a series of reforms in drug regulation and clinical trial management. These changes need to be closely evaluated to be sure that they enhance the efficacy and safety of new drugs.

## 1 Introduction

Decisions about whether to approve new active substances (drugs that have never been previously available in Canada in any form, hereafter referred to as “new drugs”) require the evaluation of a large amount of information along with a decision about what type of review procedure to use–standard (review timeline of 300 days), priority (review timeline of 180 days), or Notice of Compliance with conditions (NOC/c, conditional approval subject to the results of postmarket studies). The more complete and transparent that the information used in decision-making is, the more likely that prescribers, patients, and the public in general will support approval decisions made by Health Canada, be they positive or negative.

One source of information about the clinical trials that Health Canada evaluates is its Summary Basis of Decision (SBD) documents. The SBD is a document issued after a new drug or medical device is approved and is available on a publicly accessible website (1) that explains the scientific and benefit-to-risk information that was considered prior to approving the product. Phase I of the SBD initiative began on 1 January 2005 (2) and ran until the end of August 2012 (3). Research about that phase evaluated 161 SBDs that reported on 456 trials and found that in the majority of the SBDs at least one-third of the potential information about patient characteristics and the benefits and risks of the tested treatments was missing. The authors concluded that in its Phase I form, the SBD offered only a very modest quantity and quality of information to aid in clinical decision-making (4). Phase II of the SBDs was introduced after changes were made based on an internal evaluation of Phase I and includes all drugs approved after 1 September 2012 and has an increased focus on Health Canada’s risk to benefit analysis of the information that was assessed (5).

Drug regulation is also a dynamic process with changes in the type of drugs presented by companies for approval, for example, biologics versus small-molecule drugs; the demographics of patients enrolled in clinical trials; the structure of the trials, for example, the types of outcomes used; and the type of review process used by Health Canada.

This study examined the characteristics of new drugs that companies are submitting for approval, the quality and quantity of information that Health Canada discloses in the SBDs about the number of indications and pivotal trials per drug, the demographics of patients enrolled in the trials and trial characteristics, and the type of review that it used to approve the drugs. It examines whether there have been changes in these three types of data between when Phase II of the SBDs started until 31 March 2022, and finally, it compares the quantity of information disclosed in Phase II with that disclosed in Phase I.

## 2 Methods

### 2.1 List of new drugs

A list of all new drugs approved from 1 September 2012 to 31 March 2022 was constructed based on the annual reports from two divisions of Health Canada, the Pharmaceutical Products Directorate (PPD, previously the Therapeutic Products Directorate) and the Biologics and Radiopharmaceutical Drugs Directorate (BRDD, previously the Biologics and Genetic Therapies Directorate), which are available by emailing publications-publications@hc-sc.gc.ca. From these reports, the brand and generic names, date of approval, and type of drug (biologic or small molecule drug) were entered into an Excel file.

### 2.2 Characteristics of new drugs

The World Health Organization Collaborating Centre for Drugs Statistics Methodology was searched for each new drug to determine its Anatomic Therapeutic Chemical category at the second (of five) level (6). Since 2017, Health Canada annual reports state whether new drugs were classified as orphan drugs by either the United States (US) Food and Drug Administration (FDA) or the European Medicines Agency (EMA) (7). Previous to 2017, orphan drug status was determined from information directly from the FDA (8) and the EMA (9). Information about both characteristics was entered in the Excel file.

### 2.3 Information in the Summary Basis of Decision

The number of indications for each drug was determined from Section 7.1 of the SBD, “Clinical basis for decision”; if the information was absent from that section, then Section 1, “What was approved” was consulted, and if it was not stated in either of these SBD sections, then the Product Monograph, available at the Drug Product Database, was examined (10). If the latter was used, it was important to determine just the initial approved indication(s) as additional indications may have been subsequently approved.

Section 7.1 contains information about the pivotal trials used in making a decision to approve a drug. The following characteristics about each drug and pivotal trial were extracted from the SBDs (1): number of pivotal trials per drug, phase of each trial (1, 2, and 3, no information), number of arms per trial (1, 2, 3, and 4+, no information), randomization (yes and no, no information), controlled (placebo, active, no, and other type of control, no information), blinded (yes and no, no information), information about the number of patients per trial (individually for each trial and collectively for each drug, no information), number of patients enrolled per trial, age of patients given (yes and no), sex breakdown of patients given (yes and no), trial design (superiority, non-inferiority, single arm, and no comparison, no information), and outcome used (clinical and surrogate, no information). Information from non-pivotal trials was not examined. If a single trial had multiple phases, the highest phase was recorded. Similarly, if a single trial at times had no control and at other times was controlled, then the trial was deemed to be controlled. If a trial only had a single arm, it was assumed that it was not randomized, controlled, or blinded even if the SBD did not explicitly say so. All the information was entered in the Excel file.

### 2.4 Type of review

The type of review used for each drug–standard, priority, NOC/c, and NOC/c plus priority–was determined from the annual reports from the PPD and the BRDD and entered into the Excel file.

All the data were extracted by a single author between June 15 and 15 July 2023.

### 2.5 Data analysis

Drug characteristics, patient demographics, trial characteristics, and type of review were reported for the entire time period as either percent or median values (with interquartile range, IQR) as appropriate. The data were then broken down into three time periods depending on when the drug was approved: 1 September 2012 to 31 December 2015; 1 January 2016 to 31 December 2018; 1 January 2019 to 31 March 2022 and the three periods were compared using either chi-square or Kruskal–Wallis tests, as appropriate, with a *p* < 0.05 considered significant. The break at the end of 2015 was based on a Health Products and Food Branch strategic plan for 2016–2021, which analyzed the evolving regulatory environment and outlined planned changes in four areas: openness and transparency, collaboration, the organization and people, and innovation (11). The second breakpoint at the end of 2018 was chosen because of the release of a draft guidance on the use of accelerated reviews of drug submissions (12) and the adoption of “agile regulation” by Health Canada in mid-2019 (13).

Previous research in the United States has documented differences in clinical trial characteristics between orphan and non-orphan drugs and an association between those differences and the type of regulatory pathway used (14, 15). Therefore, both of these metrics were compared between the two types of drugs.

Because of differences in the type of information gathered and the way that it was reported in the study of Phase I of the SBDs (4) and this study, it was only possible to compare the two on the following metrics: mention of age and sex of patients, number of patients per trial, and presence or absence of a comparator. Comparisons were made using a chi-square test.

Calculations were made with Graph Pad Prism.^1^

### 2.6 Ethics and data availability

All data were publicly available and ethics approval was not required. All data extracted is available in the Supplementary material.

## 3 Results

**Figure 1:**
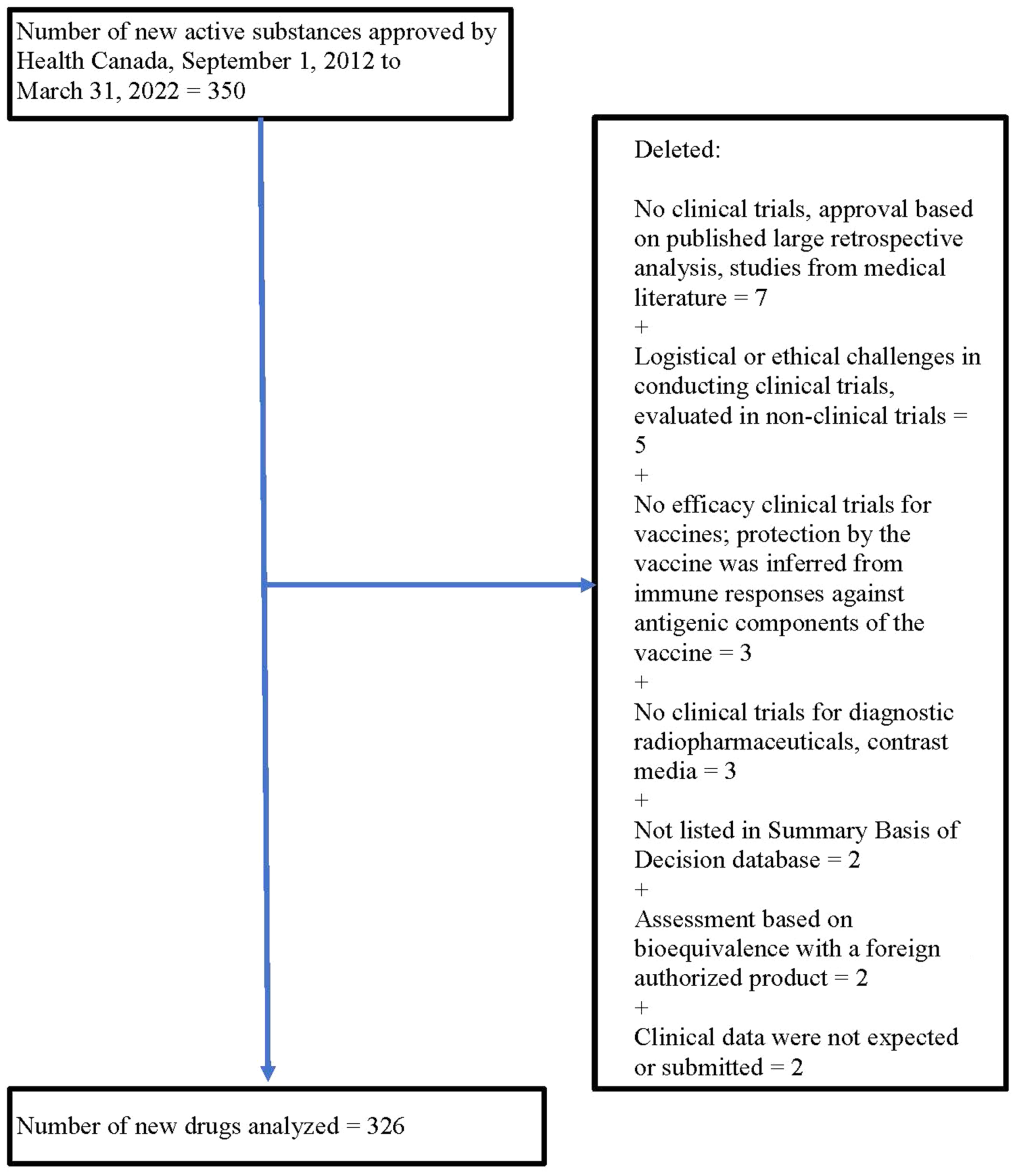
Flow chart for number of new drugs analyzed.

From 1 September 2012 to 31 March 2022, Health Canada approved 350 new drugs. Twenty-four drugs were excluded for a variety of reasons (see Figure 1), leaving 326 drugs for analysis.

### 3.1 Characteristics of new drugs

Table 1 summarizes the characteristics of the drugs. Almost two-thirds (211, 64.7%) of new drugs were small molecules. The plurality was for drugs for non-orphan diseases (156, 47.9%), 140 (42.9%) were drugs for orphan diseases (“orphan drugs”), and information about orphan status was lacking for 30 (9.2%). Over a quarter (89, 27.3%) of the drugs were antineoplastic agents, of which 67 were orphans, and no other therapeutic category comprised more than 9% of the new drugs.

**Table 1:** Characteristics of new drugs approved by Health Canada, 1 September 2012 to 31 March 2022.

Characteristic	Entire time period	1 September 2012 to 31 December 2015	1 January 2016 to 31 December 2018	1 January 2019 to 31 March 2022	Value of p*
New drugs					
Number (*n*)	326	104	105	117	
Type of drug					
Small molecule (%)	211 (64.7)	73 (70.2)	67 (63.8)	71 (60.7)	*p* = 0.3263, chi-square test
Biologic (%)	115 (35.3)	31 (29.8)	38 (36.2)	46 (39.3)	
Orphan^†^					
Yes (%)	140 (42.9)	37 (35.6)	43 (41.0)	60 (51.3)	*p* = 0.0167, chi-square test
No (%)	156 (47.9)	62 (59.6)	50 (47.6)	44 (37.6)	
No information (%)	30 (9.2)	5 (4.8)	12 (11.4)	13 (11.1)	
Therapeutic category^‡^					
Antineoplastic agents (%)	89 (27.3)	21 (20.2)	24 (22.9)	44 (37.6)	*p* = 0.1503, chi-square test
Immunosuppressants (%)	27 (8.3)	9 (8.7)	7 (6.7)	11 (9.4)	
Antivirals for systemic use (%)	23 (7.1)	8 (7.7)	9 (8.6)	6 (5.1)	
Other alimentary (%)	20 (6.1)	7 (6.7)	8 (7.6)	5 (4.3)	
All others (%)	167 (51.5)	59 (56.7)	57 (54.3)	51 (43.6)	

*Comparison of three time periods. ^†^Before 2017 as per the United States Food and Drug Administration and European Medicines Agency, 2017 onwards as per Health Canada. ^‡^World Health Organization Anatomic, Therapeutic, Chemical second level.

There was an increase in the percent of drugs with orphan status over time (*p* = 0.0167, chi-square test), rising to 51.3% in the 2019–2022 period. Antineoplastic drugs as a percent of all new drugs went from 20.2% in 2012–2015 to 37.6% in 2019–2022, but there was no statistically significant change in the distribution of therapeutic categories. The breakdown between small-molecule drugs and biologics did not change over the three time periods.

### 3.2 Information in the Summary Basis of Decision

Table 2 summarizes the trial characteristics. There was a total of 407 indications initially approved with a median of 1 (IQR 1, 1) indication per drug based on 664 pivotal trials with a median of 2 trials (IQR 1, 2) per drug. Usually, information about the pivotal trials was reported for each trial, but at times, it was reported for all trials collectively. The majority of trials were Phase 3 (69.4%), but information was lacking about the phase for 18.4%. Just over 50% of all trials had 2 arms. Over three-quarters of the trials were randomized (76.4%), and 76.2% were controlled (placebo = 46.7%, active = 24.8%, other = 4.7%) and just over 60% were blinded.

**Table 2:** Information in the Summary Basis of Decision about characteristics of pivotal trials and patient demographics used by Health Canada in approving new drugs, 1 September 2012 to 31 March 2022.

Characteristic	Entire time period	1 September 2012 to 31 December 2015	1 January 2016 to 31 December 2018	1 January 2019 to 31 March 2022	Value of p*
Indications					
Number (*n*)	407	137	133	136	
Indications per drug, median (IQR)	1 (1,1)	1 (1,1)	1 (1,1)	1 (1,1)	*p* = 0.0817, Kruskal–Wallis test
Pivotal trials					
Number	664^†^	245	223	196	
Number per drug, median (IQR)	2 (1, 2)	2 (1, 3)	2 (1, 2)	1 (1, 2)	*p* = 0.0091, Kruskal–Wallis test
Phase of trial					
1 (%)	13 (2.0)	2 (0.8)	5 (2.2)	6 (3.1)	*p* = 0.005, chi-square test
2 (%)	69 (10.4)	13 (5.3)	25 (11.2)	31 (15.8)	
3 (%)	461 (69.4)	187 (76.3)	147 (65.9)	127 (64.8)	
No information (%)	122 (18.4)	43 (17.6)	46 (20.6)	32 (16.3)	
Number of arms per trial					
1 (%)	93 (14.0)	24 (9.8)	28 (12.6)	41 (20.9)	*p* < 0.0001, chi-square test
2 (%)	334 (50.3)	116 (47.3)	109 (48.9)	109 (55.6)	
3 (%)	116 (17.5)	42 (17.1)	43 (19.3)	31 (15.8)	
4+ (%)	47 (7.1)	28 (11.4)	11 (4.9)	8 (4.1)	
No information (%)	74 (11.4)	35 (14.3)	32 (14.3)	7 (3.6)	
Randomized					
Yes (%)	507 (76.4)	200 (81.6)	165 (74.0)	143 (73.0)	*p* = 0.0001, chi-square test
No (%)	99 (14.9)	26 (10.6)	31(13.9)	46 (23.5)	
No information (%)	58 (8.7)	19 (7.6)	27 (12.1)	7 (3.6)	
Control					
Placebo (%)	310 (46.7)	134 (54.7)	88 (39.5)	88 (44.9)	*p* = 0.0019, chi-square test
Active (%)	165 (24.8)	49 (20.0)	67 (30.0)	49 (25.0)	
No (%)	125 (18.8)	42 (16.8)	36 (16.1)	47 (24.0)	
Other (%)	31 (4.7)	9 (3.7)	14 (6.3)	8 (4.1)	
No information (%)	33 (5.0)	11 (4.5)	18 (8.1)	4 (2.0)	
Blinded					
Yes (%)	413 (62.2)	177 (72.2)	131 (58.7)	105 (53.6)	*p* = 0.0006, chi-square test
No (%)	188 (28.3)	49 (20.0)	73 (32.7)	66 (33.7)	
No information (%)	63 (9.5)	19 (7.8)	19 (8.5)	25 (12.8)	
Information given about number of patients per trial					
Yes–individually for each trial (%)	459 (69.1)	161 (65.7)	136 (61.0)	162 (82.7)	*p* < 0.0001, chi-square test
Yes–collectively in all trials for each drug (%)	156 (23.5)	63 (25.7)	64 (28.7)	29 (14.8)	
No (%)	49 (7.4)	21 (8.6)	23 (10.3)	5 (2.6)	
Patients enrolled per trial					
Median (IQR)	457 (161, 818)	486 (222, 814)	460 (154, 856)	305 (107, 789)	*p* = 0.0494, Kruskal–Wallis test
Age of patients given					
Yes (%)	238 (35.8)	76 (31.0)	71 (31.8)	91 (46.4)	*p* = 0.0011, chi-square test
No (%)	426 (64.2)	169 (69.0)	152 (68.2)	105 (53.6)	
Sex breakdown given^‡^					
Yes (%)	158 (23.8)	58 (23.7)	40 (17.9)	60 (30.6)	*p* = 0.0098, chi-square test
No (%)	506 (76.2)	187 (76.3)	183 (82.1)	136 (69.4)	
Trial design					
Superiority (%)	7 (1.1)	0 (0)	3 (1.3)	4 (2.0)	*p* = 0.0019, Fisher’s exact test
Non-inferiority (%)	45 (6.9)	12 (4.9)	22 (9.9)	11 (5.6)	
Single arm only, no comparison (%)	115 (17.2)	30 (12.2)	35 (15.7)	50 (25.5)	
No information (%)	497 (74.8)	203 (82.9)	163 (70.1)	131 (66.8)	
Outcome used					
Clinical (%)	166 (50.9)	56 (53.8)	56 (53.3)	54 (46.2)	*p* = 0.4771, chi-square test
Surrogate (%)	159 (48.8)	48 (46.2)	49 (46.7)	62 (53.0)	
No information (%)	1 (0.3)	0	0	1 (0.8)	

*Comparison of three time periods. ^†^Number of pivotal trials missing for three drugs. ^‡^Number of trials for products used exclusively by one sex: total (64), 1 September 2012–31 December 2015 (16), 1 January 2016–31 December 2018 (15), 1 January 2019–30 April 2022 (28).

There was information about the number of patients enrolled per individual trial for 69.1% of trials and information about the number of patients in all trials per drug an additional 23.5% times. The median (IQR) number of patents per trial was 457 (161, 818). Only a minority of trials gave the age of patients (35.8%) and the sex breakdown (23.8%). There was no information about whether the trial was designed as a superiority or non-inferiority one in 74.8% of cases. Finally, the outcome used in the trials was almost evenly split between clinical (50.9%) and surrogate (48.8%).

Over the time of the study, the number of indications per drug decreased (*p* = 0.0817, Kruskal–Wallis test) as did the number of pivotal trials per drug (*p* = 0.0091, Kruskal–Wallis test). The percent of Phase 3 trials dropped from 76.3% in 2012–2015 to 64.8% in 2019–2022 (*p* = 0.005, chi-square test), and in each time period, there was no information about the phase in over 15% of trials. The distribution of the number of arms per trial skewed toward a smaller number of arms (*p* < 0.0001, chi-square test) with single-arm trials increasing from 9.8% in 2012–2015 to 20.9% in 2019–2022. There was also a decrease in the percent of trials that were randomized, controlled, and blinded (*p* = 0.0001, *p* = 0.0019, *p* = 0.0006, respectively, chi-square test).

Reporting in the SBDs about the percent of trials giving the number of patients increased (*p* < 0.0001, chi-square test), whereas the median number of patients in each trial decreased (*p* = 0.0494, Kruskal–Wallis test). More trials provided the age of the patients (*p* = 0.0011, chi-square test) and the sex breakdown (*p* = 0.0098, chi-square test) although even in the final time period (2019–2022) only a minority of trials gave each type of information. Information about age was highly variable, reported as means, medians, range, and greater than or less than a certain value. There was an increase in the percent of trials with information about trial design (*p* = 0.0019, chi-square test), however, in all three time periods the majority of trials lacked information about design. There was no change in the percent of trials using a clinical or surrogate outcome.

### 3.3 Comparison of clinical trial characteristics between orphan and non-orphan drugs

Clinical trials of orphan drugs differed from those of non-orphan drugs in all metrics that were measured (Table 3). Phase 2 trials were more likely to be used as the basis for their approval, they had a smaller number of trial arms (*p* = 0.0001, chi-square test), they had a statistically significant smaller number of enrolled patients (*p* < 0.0001, Mann–Whitney test), they used surrogate outcomes more often (*p* < 0.0001, Fisher’s exact test), they were less likely to be randomized (*p* < 0.0001, chi-square test), they were less likely to be controlled (*p* < 0.0001, chi-square test) and less likely to be blinded (*p* < 0.0001, chi-square test).

**Table 3:** Characteristics of trials for orphan and non-orphan drugs and type of review used, 1 September 2012–31 March 2022.

Characteristic	Orphan drugs	Non-orphan drugs	Value of *p*
Total number	140	156	
Pivotal trials			
Number per drug, median (IQR)	1 (1,2)	1 (1,3)	*p* < 0.0001, Mann–Whitney test
Phase of trial			
1 (%)	4 (2.9)	5 (3.2)	*p* < 0.0001, chi-square test
2 (%)	39 (27.9)	13 (8.3)	
3 (%)	86 (61.4)	115 (73.7)	
No information (%)	11 (7.9)	23 (14.7)	
Number of arms per trial			
1 (%)	36 (25.7)	13 (8.3)	*p* < 0.0001, chi-square test
2 (%)	78 (55.7)	85 (54.5)	
3 (%)	14 (10.0)	30 (19.2)	
4+ (%)	7 (5.0)	12 (7.7)	
No information (%)	5 (3.6)	16 (10.3)	
Patients enrolled per trial			
Median (IQR)	170 (93, 352)	824 (477, 1,510)	*p* < 0.0001, Mann–Whitney test
Outcome used			
Clinical (%)	91 (65.0)	63 (40.4)	*p* < 0.0001, Fisher’s exact test
Surrogate (%)	48 (34.3)	93 (59.6)	
No information (%)	1 (0.7)	0 (0)	
Randomized			
Yes (%)	97 (69.3)	137 (87.8)	*p* < 0.0001, chi-square test
No (%)	41 (29.3)	14 (9.0)	
No information (%)	2 (1.4)	5 (3.2)	
Control			
Placebo (%)	53 (37.9)	93 (59.6)	*p* < 0.0001, chi-square test
Active (%)	28 (20.0)	37 (23.7)	
No (%)	44 (31.4)	20 (12.8)	
Other (%)	12 (8.6)	5 (3.2)	
No information (%)	3 (2.1)	1 (0.6)	
Blinded			
Yes (%)	56 (40.0)	116 (74.4)	*p* < 0.0001, chi-square test
No (%)	74 (52.9)	26 (16.7)	
No information (%)	10 (7.1)	14 (9.0)	

### 3.4 Type of review

A standard review was used in the evaluation of 62.3% of drugs, and a priority review was used one-quarter of the time (Table 4). Over the entire time period, the percent of drugs with a standard review dropped from 70.2 to 56.4%, while the percent of NOC/c reviews increased from 8.7 to 16.2%, but the change in the distribution of review types was not statistically significant (*p* = 0.1719, chi-square test).

**Table 4:** Type of review used by Health Canada.

Type of review	Entire time period, *n* = 326 (%)	1 September 2012 to 31 December 2015, *n* = 104 (%)	1 January 2016 to 31 December 2018, *n* = 105 (%)	1 January 2019 to 31 March 2022, *n* = 117 (%)	Value of p*
Standard	203 (62.3)	73 (70.2)	63 (60.0)	66 (56.4)	*p* = 0.1719, chi-square test
Priority	81 (24.8)	22 (21.2)	29 (27.6)	30 (25.6)	
Notice of Compliance with conditions	40 (12.3)	9 (8.7)	12 (11.4)	19 (16.2)	
Notice of Compliance with conditions + priority^†^	3 (0.9)	0	1 (1.0)	2 (1.7)	

*Comparison of three time periods. ^†^Notice of Compliance with conditions + priority combined with Notice of Compliance with conditions for chi-square test.

The distribution of review types was statistically significantly different between orphan drugs, non-orphan drugs, and drugs for which there was no information about orphan status, with orphan drugs more likely to receive either a NOC/c or a NOC/c + priority review (*p* < 0.0001, chi-square test; Table 5).

**Table 5:** Distribution of review types for orphan and non-orphan drugs, 1 September 2012 to 31 March 2022.

Orphan drug status	Total number of drugs (%)	Standard review (%)	Priority review (%)	Notice of Compliance with conditions + Notice of Compliance with conditions and priority (%)	Value of *p*
Orphan	140 (42.9)	53 (37.9)	56 (40.0)	31 (22.1)	*p* < 0.0001, chi-square test
Non-orphan	156 (47.9)	122 (78.2)	23 (14.7)	11 (7.1)	

### 3.5 Comparison of the quantity of information in phases I and II of SBD

There was no difference in the percent of trials where the age of the patients was given (*p* = 0.3401, chi square test), and in both phases, the SBDs provided this information only for a minority of trials (Table 6). The SBDs gave more information about the sex of patients and the number of patients per trial in Phase II, but in both phases, data about sex were only available for a minority of trials. Finally, the SBDs reported a lower percent of trials in Phase II using any type of comparator than in Phase I (*p* < 0.0001, chi-square).

**Table 6:** Comparison of information about trials in Summary Basis of Decision Phase I and Phase II.

Phase I*		Phase II		Comparison
Age of patients, *n* (percent)				
Yes^†^	No	Yes	No	*p* = 0.3401, chi-square test
150 (32.9)	306 (67.1)	238 (35.8)	426 (64.2)	
Sex of patients, *n* (percent)				
Yes^†^	No	Yes	No	*p* = 0.0037, chi-square test
75 (16.4)	381 (83.6)	158 (23.8)	506 (76.2)	
Number of patients in trial, *n* (percent)				
Yes^†^	No	Yes^‡^	No	*p* = 0.0056, chi-square test
399 (87.5)	57 (12.5)	615 (93.6)	49 (7.4)	
Comparator used, *n* (percent)				
Yes^†^^§^	No	Yes^¶^	No^Ɨ^	*p* < 0.0001, chi-square test
443 (97.1)	13 (2.9)	506 (76.2)	158 (23.8)	

*Data from Habibi and Lexchin (4). ^†^Information described completely or partially. ^‡^Number of patients in individual trials + number of patients in all trials for each drug. ^§^Placebo + active. ^¶^Placebo + active + other. ^Ɨ^No + no information.

## 4 Discussion

### 4.1 Change in regulatory standards

This study analyzed 326 new drugs for 407 indications approved by Health Canada over the period 1 September 2012 to 31 March 2022. There were more small-molecule drugs approved overall, and there was no difference in the distribution between biologics and small-molecule drugs over time and the same was true in the distribution of therapeutic categories.

However, there was a decrease in the number of pivotal trials per drug perhaps reflecting the trend over time in the approval of more orphan drugs. This latter trend coincided with an increase in single-arm trials from 9.8% in 2012 to 2015 to 20.9% in 2019–2022 and a decrease in the percent of trials that were randomized, blinded, controlled, and in the use of Phase 3 trials. Orphan drugs were also more likely to receive either a priority or an NOC/c review compared to non-orphan drugs; an NOC/c review typically relies on preliminary Phase 2 data with the drug’s efficacy needing to be confirmed through postmarket studies (17). The median number of patients per trial has also declined from 486 in 2012 to 2015 to 305 in 2019–2022.

These results could be due to regulatory standards becoming less rigorous over time, they could reflect an adaptation to the increase in the number of orphan drugs being submitted or a combination of both reasons. The NOC/c program, under which 31 of the 140 orphan drugs were approved, is designed to facilitate approvals based on fewer trials, shorter trials, or trials using surrogate markers. Approvals can be made without requiring costly and time-consuming randomized, double-blinded, controlled trials. Designing trials for orphan drugs presents a number of challenges when small patient numbers limit power. Small numbers of patients may translate into single-arm trials; if there is no existing therapy to give to a control group, enrolling a placebo group may be considered unethical for debilitating or fatal diseases. In some cases, a change to more flexible regulatory standards may hasten the availability of orphan drugs for patients and result in meaningful benefits. Similarly, the perceived need to enhance access to potentially beneficial drugs may result in more approvals through priority and NOC/c pathways. Finally, the nature of therapeutics is evolving with novel products such as those based on advanced cell and gene therapy increasingly being sent to drug regulatory agencies for approval. These products require the use of customized regulatory requirements that “allow the agility and flexibility necessary to determine the appropriate oversight of innovative health products” (18).

However, the question of the degree to which changes in regulatory standards are promoting the introduction of products, such as orphan drugs, that offer significant therapeutic gains needs to be investigated. In this regard, an analysis of the use of the NOC/c pathway for oncology drugs by McPhail et al. (17) has questioned whether it is being appropriately utilized because of the lack of definitional clarity for assessing which drugs are eligible for inclusion. Other researchers have pointed out that up to the end of 2017, the NOC/c pathway had been used for 89 new drugs of which 78 had a therapeutic evaluation, and of these, 54 of 78 drugs offered only minimal or no therapeutic gains compared to existing products (19).

### 4.2 Change in the transparency of information in SBDs

At the same time as regulatory standards have been changing, the SBDs are becoming more transparent in the amount of information in some categories including the number of patients per trial and the age and sex of patients in the trials, although in the latter two categories the information is still more likely to be absent than present. Phase II SBDs are also more transparent than Phase I ones in disclosing the sex of patients and the number of patients per trial. There was a lower percent of trials in Phase II using comparators than Phase I and that trend continued through the three Phase II time periods. The SBDs analyzed in the present study are overwhelmingly unlikely to disclose if trials were designed as superiority or non-inferiority ones.

### 4.3 Comparison with similar research on the Food and Drug Administration

The results reported here reflect similar changes seen in the assessment of clinical trials evaluated by the FDA. Between 2005 and 2012, nearly all trials were randomized, double-blinded, and used either an active or placebo comparator, and 51.2% used either clinical or clinical scale outcomes (14). A later study comparing three time periods, 1995–1997, 2005–2007, and 2015–2017 found the proportion of therapeutic approvals receiving an orphan designation increased from 12.7% in 1995–1997 to 38.1% in 2015–2017. The proportion of indications supported by at least two pivotal trials decreased over time and the proportion of indications supported by only single-arm pivotal trials increased (15). The authors of the second study also commented that the evidence supporting approvals had become less rigorous in some ways, although the authors of both studies also noted that regulatory standards needed to become more flexible to accommodate the increase in the number of oncology and orphan drugs being submitted for approval.

### 4.4 Ongoing reforms of the drug regulatory system of Health Canada

Health Canada has recently embarked on a series of reforms termed “agile” drug regulation, which it defines as amendments designed to “keep pace with innovation and facilitate access to advanced treatments and promising therapies, while continuing to ensure authorized drugs…are safe, effective, and subject to appropriate oversight” (20). In conjunction with the agile regulatory reforms is a plan to modernize Health Canada’s clinical trials framework and “streamline processes toward greater efficiency and clarity, and align with international best practices regarding oversight and public access to information” (21).

Health Canada maintains that agile regulation will continue to ensure authorized drugs are safe, effective, and subject to appropriate oversight (20), but in its 2018 Budget, the federal government was also promoting a regulatory reform agenda that would support innovation and business investments and would target regulatory requirements and practices that were bottlenecks to innovation and growth in Canada (13). Critics of agile regulation have charged that it would allow companies to bring drugs to market up to 6 months earlier than is currently allowed and that drug approvals would require fewer premarket clinical trials “as long as firms continue studying their drugs’ effectiveness after they are already in use” (16).

At this point, Health Canada has not presented any evidence that its modernization initiatives will result in products with clinically meaningful benefits reaching patients sooner without jeopardizing patient safety. The results of this present study raise questions about a possible decline in regulatory standards that may be accelerated by recent initiatives of Health Canada.

### 4.5 Limitations

The data were extracted by a single person which could have introduced either transcription errors or errors in the interpretation of the data about trials, for example, whether they used a surrogate or clinical outcome. The absence of information in the SBD does not mean that the information was lacking in the new drug application that companies submitted to Health Canada. The sometimes haphazard way that data were reported in the SBD, for example, reporting information about trials collectively, rather than by individual trial made gathering the data difficult at times. The date of receipt of marketing authorization sometimes varied between Health Canada databases (Notice of Compliance database, Summary Basis of Decision database, annual reports from PPD and BRDD), and in these cases, the annual reports were taken as definitive. Health Canada’s analysis of the data in the pivotal trials was not examined to determine whether approval of the drugs was justified. Changes to the traditional clinical trial methodology are necessary to accommodate the unique circumstances of orphan drugs. However, the resources to evaluate whether the changes were appropriate for all of the orphan drugs reviewed were beyond the capacity of this study.

## 5 Conclusion

This study evaluated data involving the approval of 326 new drugs by Health Canada between 1 September 2012 and 31 March 2022 and the 664 clinical trials supporting those approvals. It found a change in regulatory standards that may be due to them becoming less rigorous, because of an adaptation to the number of orphan drugs being submitted or a combination of both reasons. At the same time, there was also an increase in transparency in some elements of the SBDs. Changes in drug regulation recently undertaken by Health Canada will further affect standards and these reforms need to be closely evaluated to ensure that they enhance the efficacy and safety of new drugs.

## Data availability statement

The original contributions presented in the study are included in the article/Supplementary material, further inquiries can be directed to the corresponding author.

## Author contributions

JL: Conceptualization, Data curation, Formal analysis, Methodology, Writing – original draft, Writing – review & editing.

## Footnotes

^1^version 10.02, www.graphpad.com
